# Lactoferrin for iron-deficiency anemia in children with inflammatory bowel disease: a clinical trial

**DOI:** 10.1038/s41390-022-02136-2

**Published:** 2022-06-09

**Authors:** Doaa El Amrousy, Dalia El-Afify, Abdallah Elsawy, Mai Elsheikh, Amr Donia, Mohammed Nassar

**Affiliations:** 1 Pediatric Department, Faculty of Medicine, Tanta University, Tanta, Egypt; 2 Department of Clinical Pharmacy, Faculty of Pharmacy, Tanta University, Tanta, Egypt; 3 Department of Internal Medicine, Faculty of Medicine, Tanta University, Tanta, Egypt

## Abstract

**Background:**

Iron-deficiency anemia (IDA) is common in children with inflammatory bowel disease (IBD); however, oral iron supplements are commonly associated with poor compliance due to gastrointestinal side effects. We compared the effect of lactoferrin versus oral ferrous sulfate for the treatment of IDA in children with IBD.

**Methods:**

Ninety-two IBD children with IDA were included but only 80 children completed the study and they were randomized into two groups: ferrous sulfate group (*n* = 40) who received ferrous sulfate 6 mg/kg/day for 3 months and lactoferrin group (*n* = 40) who received lactoferrin 100 mg/day for 3 months. Complete blood count, serum iron, total iron-binding capacity (TIBC), transferrin saturation (TS), serum ferritin, interleukin-6 (IL-6), and hepcidin 25 were measured before and after the treatment.

**Results:**

Hemoglobin (Hb), mean corpuscular volume, serum iron, TS, and serum ferritin significantly increased, while TIBC decreased significantly after the administration of either ferrous sulfate or lactoferrin compared to their baseline data. In addition, lactoferrin significantly increased Hb, serum iron, TS, and serum ferritin compared to ferrous sulfate. Moreover, lactoferrin significantly decreased IL-6 and hepcidin levels.

**Conclusion:**

Lactoferrin is a promising effective treatment with fewer side effects than oral elemental iron in children with IBD and IDA.

**Clinical trial registration:**

The study was registered at www.pactr.org (PACTR202002763901803).

**Impact:**

- Iron-deficiency anemia (IDA) in children with inflammatory bowel disease (IBD) is treated with oral iron therapy; however, oral iron supplements are commonly associated with poor compliance due to gastrointestinal side effects.
- To the best of our knowledge, our study was the first in pediatrics that compared the effect of lactoferrin versus oral ferrous sulfate as an iron supplement for the treatment of IDA in children with IBD.
- We found that lactoferrin is a promising effective treatment with fewer side effects than oral elemental iron in children with IBD and IDA.

## Introduction

Anemia is considered the most common extra-intestinal complication of inflammatory bowel disease (IBD) in children. Anemia affects growth, quality of life, and neurocognitive development in children with IBD.^1^ The pathophysiology of anemia in IBD is multifactorial and it involves a combination of both iron-deficiency anemia (IDA) and anemia of chronic disease (ACD).^2^

Iron deficiency in IBD is related to several factors including chronic gastrointestinal bleeding, mucosal inflammation and sloughing of gastrointestinal lining cells, decreased iron absorption, and inadequate iron intake due to malnutrition associated with dietary restrictions and poor appetite.^3^ ACD is also involved in alterations in iron absorption, distribution, and transport in addition to decreased erythropoiesis.^2–4^ IDA in children is commonly treated with oral and intravenous iron therapy; however, oral iron supplements are commonly associated with poor compliance due to gastrointestinal side effects such as abdominal pain, nausea, and vomiting.^5^

Lactoferrin is a multifunctional iron-binding glycoprotein that can modulate immunity, inflammation, and enhance iron absorption.^6^ Lactoferrin has been reported as an effective therapy in the treatment of anemia in both pediatrics and adults.^7–9^

Several studies assessed the efficacy of lactoferrin in neonates, infants, and children on gut health and neonatal sepsis and found that it is effective with almost no adverse effects.^10,11^ The efficacy of lactoferrin in the treatment of IBD-related anemia was not previously studied. Therefore, the aim of this study was to compare the effect of lactoferrin versus oral ferrous sulfate as an iron supplement in the treatment of IDA in children with IBD.

## Patients and methods

This randomized clinical trial was conducted on 80 children with IBD-related IDA who were recruited from the outpatient clinic of the Pediatric Department, Tanta University Hospitals during the period from February 2020 to August 2021 after the approval of the ethical committee of the Faculty of Medicine, Tanta University. The study was registered at www.pactr.org (PACTR202002763901803). Informed consent was obtained from the parents of all patients.

Inclusion criteria: children with IBD either ulcerative colitis (UC) or Crohn’s disease (CD) in remission with IDA aged 5–18 years were included. Patients were diagnosed according to the European Society of Pediatric Gastroenterology, Hepatology, and Nutrition revised Porto criteria.^12^

IDA in IBD was defined as a hemoglobin (Hb) level lower than the normal range for age and sex. Lower limits for Hb values specified by the World Health Organization were less than 11.5 g/dl for children 5–11 years, less than 12 g/dl for children 12–14 years and females of 15 years or more, and less than 13 g/dl for males of 15 years or more.^13^

Our patients were in remission at the start of the study. The disease activity in CD group was assessed using Pediatric Crohn’s disease Activity Index (PCDAI) with scores ranging from 0 to 100. PCDAI score <10 is consistent with remission.^14^ The disease activity in UC group was assessed using Pediatric Ulcerative Colitis Activity Index (PUCAI). The PUCAI score ranges from 0 to 85 and score <10 denotes remission.^15^

Exclusion criteria: children with other chronic diseases (such as chronic liver or renal disease, immune-mediated inflammatory disorders, and malignancy), children with chronic or current infection, children with a history of allergy to cow’s milk, or recent administration of iron supplement or blood transfusion within the previous 3 months were excluded from our study.

The patients were randomized into two groups:

Ferrous sulfate group: 46 children with IBD and IDA who received ferrous sulfate in a dose of 6 mg/kg/day for 3 months.

Lactoferrin group: 46 children with IBD and IDA who received bovine lactoferrin (Pravotin sachet, Hygint, Egypt) at a dose of 100 mg/day for 3 months.

Patients were randomized to the study groups using a random block size of six through computer-generated random numbers in a 1:1 ratio. The randomization was performed by an independent statistician. Sealed opaque envelopes with sequential numbers were used to perform allocation concealment. The sealed opaque envelope was opened after signing the consent and the patient was enrolled into the respective group.

Detailed medical history was taken by the treating physicians and blood samples were obtained for C-reactive protein (CRP) level measurement using the quantitative turbidimetric method according to commercial kits (Spinreact, Ctra Santa Colona, Spain).

Routine investigations of IDA were carried out at the baseline and after 3 months of treatment including complete blood count, Hb level, mean corpuscular volume (MCV), serum iron, and total iron-binding capacity (TIBC) that were measured using commercial kits (Spectrum Diagnostics, Cairo, Egypt). Transferrin saturation (TS) was calculated as follows TS = (serum iron / total iron-binding capacity) × 100%. TS of less than 16% was considered abnormal.^13^

Serum ferritin was measured using commercial enzyme-linked immunosorbent assay (ELISA) assay kits (RayBiotech Inc., Norcross, Georgia). Serum ferritin levels less than 30 ng/ml were considered consistent with IBD-related IDA in the absence of inflammation and disease activity.^16^ In addition, serum interleukin-6 (IL-6) and hepcidin 25 were measured using commercially available ELISA kits (RayBiotech Inc., Norcross, Georgia and DRG International Inc., New Jersey, respectively).

All laboratory investigations were measured for all patients at baseline and after 3 months of treatment. The parents were instructed to record any side effects during the treatment period. We followed the patients through face-to-face meetings every month to provide the study materials and to take the used packages of drugs to ensure compliance with treatment.

The primary outcome was the change in Hb level after lactoferrin and ferrous sulfate treatment in children with IBD. The secondary outcomes were to assess serum ferritin, IL-6, and side effects of treatment in both groups after 3 months of treatment.

### Statistical analysis

Sample size of 38 IBD patients in each group was required to achieve the power of 90% with alpha = 0.05 to detect a difference of 2 g/dl in the mean Hb level using G* power software. Data analysis was carried out using SPSS software statistical computer package version 22. Categorical data were presented in the form of number and percentage, while quantitative data were presented in the form of mean and standard deviation if normally distributed. Normality of data was checked using the Shapiro–Wilk test. Qualitative data were compared using *χ*^2^ test, while a comparison between means of quantitative data of the two groups was carried out using Student’s *t*-test. Paired *t*-test was used for comparison of quantitative data within the same group at baseline and after 3 months. *P* value of less than 0.05 was considered statistically significant.

## Results

**Fig. 1:**
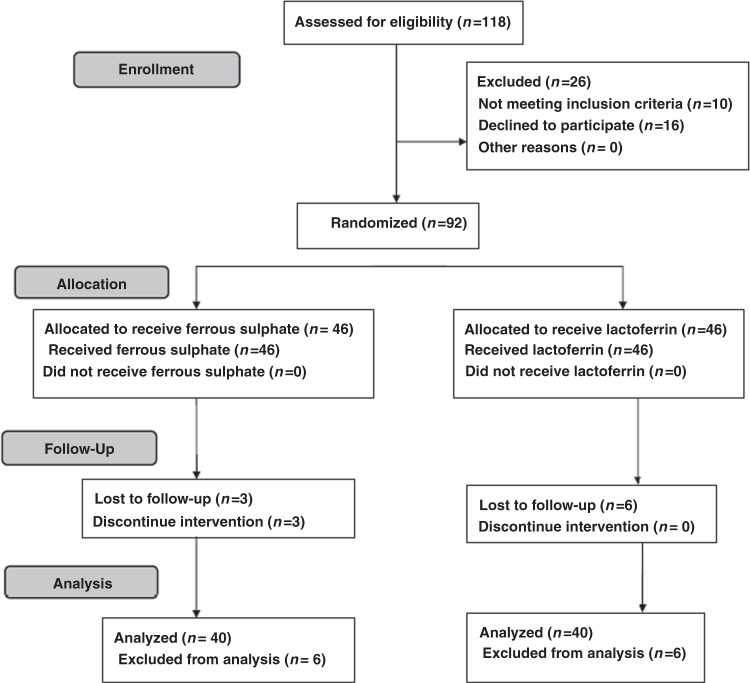
Flow diagram of the study showing details of enrollment, allocation, follow up, and analysis of the patients.

Ninety-two patients were included in the study; 46 patients in each group. Six patients were excluded from analysis in the ferrous sulfate group; three discontinued the treatment due to its side effects and we lost three patients during follow-up. Moreover, six patients were lost during follow-up in the lactoferrin group. So, only 80 patients completed the study; 40 patients in the ferrous sulfate group and 40 patients in the lactoferrin group. The study flow was summarized in Fig. 1. The study included 80 children with IBD and IDA with mean age 11.2 ± 2.4 years; 56 patients with CD and 24 patients with UC with mean disease duration 2 ± 1.1 years. Table 1 presents the baseline data of both groups and shows that there was no significant difference between the two groups as regards age, sex, type, and duration of IBD. Mean Hb, MCV, serum iron, TIBC, TS, serum ferritin, CRP, IL-6, and hepcidin 25 levels before treatment were comparable in both groups as shown in Table 1.

**Table 1:** The baseline, clinical, and laboratory data of patients of both groups.

Parameters	Ferrous sulfate group (*n* = 40)	Lactoferrin group (*n* = 40)	*P* value
Age (years)	11.4 ± 2.3	11.1 ± 2.5	0.612
Sex (male/female)	16/24	18/22	0.745
CD/UC	29/11	27/13	0.749
Duration of the disease (years)	2 ± 1.12	1.95 ± 1.01	0.762
PCDAI	8.5 ± 1.2	8 ± 1.5	0.833
PUCAI	7.8 ± 1.9	8.1 ± 1.4	0.711
Hb (g/dl)	9.2 ± 1.6	9.1 ± 1.2	0.596
MCV (fl)	72.3 ± 6.3	73.6 ± 5.2	0.64
CRP (mg/l)	4.3 ± 1.7	3.9 ± 0.7	0.837
Serum iron (µg/dl)	38.8 ± 3.8	38.6 ± 3.8	0.838
TIBC (µg/dl)	392 ± 33	398 ± 28	0.367
Transferrin saturation (%)	10 ± 1.6	9.8 ± 1.56	0.546
Serum ferritin (ng/ml)	21.3 ± 6	20.4 ± 6.5	0.537
IL-6 (pg/ml)	14 ± 3.2	13.4 ± 3.8	0.436
Hepcidin 25 (ng/ml)	32 ± 8.6	33 ± 8.8	0.6

*CD* Crohn’s disease, *UC* ulcerative colitis, *PCDAI* Pediatric Crohn’s disease Activity Index, *PUCAI* Pediatric Ulcerative Colitis Activity Index, *Hb* hemoglobin, *MCV* mean corpuscular volume, *TIBC* total iron-binding capacity, *CRP* C-reactive protein, *IL-6* interleukin-6.

After 3 months of treatment mean Hb, MCV, serum iron, TS, and serum ferritin significantly increased, while TIBC significantly decreased compared to their respective baseline data in both ferrous sulfate and lactoferrin groups as presented in Table 2. However, lactoferrin group had significantly higher mean Hb (*P* < 0.001), serum iron (*P* < 0.001), TS (*P* = 0.008), and serum ferritin (*P* = 0.006) compared to ferrous sulfate group after 3 months of treatment.

**Table 2:** Changes in laboratory parameters before and after 3 months of treatment in ferrous sulfate and lactoferrin groups.

Parameter	Ferrous sulfate group		Lactoferrin group		*P* value
	Before treatment	After treatment	Before treatment	After treatment	
Hb (g/dl)	9.2 ± 1.6	10.8 ± 0.49*	9.1 ± 1.2	11.9 ± 1.7*	0.01
MCV (fl)	72.3 ± 6.3	76.2 ± 3.2*	73.6 ± 5.2	75.8 ± 3.3*	0.606
Serum iron (µg/d)	38.8 ± 3.8	44.7 ± 3.9*	38.6 ± 3.8	49.5 ± 3.9*	0.001
TIBC (µg/dl)	392 ± 33	283 ± 38*	398 ± 28	286 ± 27*	0.7
Transferrin saturation (%)	10 ± 1.6	16 ± 1.3*	9.8 ± 1.6	17.5 ± 1.5*	0.008
Serum ferritin (ng/ml)	21.3 ± 6	32.2 ± 5.4*	20.4 ± 6.5	38.4 ± 6.1*	0.006
IL-6 (pg/ml)	14 ± 3.2	14.2 ± 2.9	13.4 ± 3.8	9.7 ± 2.5*	0.001
Hepcidin 25 (ng/ml)	32 ± 8.6	30 ± 7.4	33 ± 8.8	22 ± 6.2*	<0.001

*Hb* hemoglobin, *MCV* mean corpuscular volume, *TIB* total iron-binding capacity, *IL-6* interleukin-6.

*Significant between before and after treatment within the same group.

*P* value: difference between after treatment values of both groups.

While there was a non-significant change in IL-6 and hepcidin levels after 3 months of ferrous sulfate treatment, the administration of lactoferrin for 3 months significantly decreased both IL-6 and hepcidin levels compared to baseline data as well as the after-treatment values in the ferrous sulfate group (Table 2).

Side effects were closely monitored throughout the study and the following observations were made, 18 patients in the ferrous sulfate group (46.2%) experienced gastrointestinal side effects including abdominal pain, diarrhea, nausea, and vomiting while abdominal discomfort was reported in only one patient in the lactoferrin group (2.5%). None of the side effects necessitate the discontinuation of the treatment.

## Discussion

Oral iron therapy is the standard, easily administered, and cheap treatment for the management of pediatric IBD-associated IDA.^2–4^ However, it has several limitations including gastrointestinal side effects, poor compliance, and impaired iron absorption due to inflammation.^17^ In addition, animal studies proposed that oral elemental iron may catalyze reactions that generate oxygen-free radicals that can exacerbate inflammation and worsen the symptoms of IBD.^18^ In the present study, we compared the efficacy and the safety of lactoferrin and oral ferrous sulfate in treatment of IDA in children with IBD.

Our results revealed that the administration of either ferrous sulfate or lactoferrin significantly increase hematological parameters including Hb, MCV, serum iron, TS, and serum ferritin and significantly decreased TIBC compared to their baseline data. In addition, lactoferrin significantly increased Hb, serum iron, TS, and serum ferritin compared to ferrous sulfate. These results are in agreement with the results of previous studies in adults that reported that lactoferrin administration significantly increased Hb, serum iron, and serum ferritin compared to oral iron in pregnant and non-pregnant women^8,9,19^ and in agreement with Chen et al. that reported that lactoferrin fortified formula significantly increased Hb level compared to the iron-fortified formula in anemic infants.^7^

Lactoferrin is a glycoprotein that binds to two iron ions with high affinity and increases iron absorption. Lactoferrin enhances the uptake of iron by intestinal cells as the lactoferrin molecule enters the intestinal cell through its own receptor then iron is released from lactoferrin inside the intestinal cell and transported to the circulation via transferrin.^20^

Iron hemostasis is the balance between iron in the tissues and in the blood and is regulated by ferroportin that is an iron exporter protein that mediates the efflux of iron from enterocytes and storage tissue to blood and hepcidin. Hepcidin is a peptide synthesized by hepatocytes and increased in cases of iron overload. Hepcidin binds to ferroportin promoting its degradation and preventing iron absorption and transport from storage tissue. Inflammation associated with IBD involves the increase in inflammatory cytokines such as IL-6. IL-6 increases gene transcription of hepcidin and decreases the gene expression of ferroportin and therefore decreases intestinal iron absorption.^21,22^

In the present study lactoferrin significantly decreased IL-6 level compared to baseline data and to ferrous sulfate group and this is in accordance with Paesano et al. who reported that lactoferrin significantly decreased IL-6 in pregnant women with anemia 18. Lactoferrin may decrease the level of IL-6 due to its ability to regulate the transcription of inflammatory genes.^23^ The decrease in IL-6 may lead to downregulation of hepcidin and upregulation of ferroportin synthesis thus improving iron absorption and hemostasis.

Gastrointestinal side effects of oral iron are related to the unabsorbed iron fraction that remains in the gut lumen.^24^ In our study lactoferrin showed lower incidence of side effects compared to ferrous sulfate that may be due to the incorporation of iron in the lactoferrin molecule in the form of lactoferrin–iron complex and iron is only released inside enterocytes.

Our study reveals significant improvement in Hb, serum iron, TS, and serum ferritin in IBD children with IDA receiving lactoferrin compared to those receiving ferrous sulfate probably due to an increase in iron absorption, decreasing IL-6 and improving iron absorption and hemostasis through modulation of hepcidin and ferroportin. Moreover, lactoferrin has a lower incidence of gastrointestinal side effects and thus better patient compliance compared with ferrous sulfate.

Limitation of the study: relatively small sample size and short duration of follow-up, so further clinical trial on larger sample size with longer follow-up is required to confirm our results.

## Conclusion

Lactoferrin is a promising effective treatment with fewer side effects than oral elemental iron in children with IBD and IDA.

## Supplementary information

CONSORT 2010 Checklist

## Data Availability

The data of this research are available from the corresponding author upon reasonable request.
